# Supplementary material for: Intertwined Carbon Nanotubes and Ag Nanowires Constructed by Simple Solution Blending as Sensitive and Stable Chloramphenicol Sensors
Source: Sensors (Basel). 2021 Feb 9;21(4):1220. doi: 10.3390/s21041220 (PMC7915990; doi:10.3390/s21041220)
Supplement: Supplementary file 1 [file sensors-21-01220-s001.pdf]

Supplementary Figures

Intertwined Carbon Nanotubes and Ag Nanowires Constructed by Simple Solution Blending as Sensitive and Stable Chloramphenicol Sensors

Yangguang Zhu [1,2], Xiufen Li [1,*], Yuting Xu [3], Lidong Wu [4], Aimin Yu [5], Guosong Lai [6], Qiuping Wei [7], Hai Chi [8], Nan Jiang [2,9], Li Fu [3], Chen Ye [2,9,*] and Cheng-Te Lin [2,9,*]

1 Laboratory of Environmental Biotechnology, School of Environmental and Civil Engineering, Jiangnan University, Wuxi 214122, China;
2 Key Laboratory of Marine Materials and Related Technologies, Zhejiang Key Laboratory of Marine Materials and Protective Technologies, Ningbo Institute of Materials Technology and Engineering (NIMTE), Chinese Academy of Sciences, Ningbo 315201, China;
3 College of Materials and Environmental Engineering, Hangzhou Dianzi University, Hangzhou 310018, China;
4 Key Laboratory of Control of Quality and Safety for Aquatic Products, Chinese Academy of Fishery Sciences, Beijing, 100141, China;
5 Department of Chemistry and Biotechnology, Faculty of Science, Engineering and Technology, Swinburne University of Technology, Hawthorn VIC 3122, Australia;
6 Department of Chemistry, Hubei Normal University, Huangshi 435002, China;
7 School of Materials Science and Engineering, Central South University, Changsha 410083, China;
8 East China Sea Fisheries Research Institute, Chinese Academy of Fishery Sciences, Shanghai 200090, China;
9 Center of Materials Science and Optoelectronics Engineering, University of Chinese Academy of Sciences, Beijing 100049, China
* Correspondence: xfli@jiangnan.edu.cn (X.L); yechen@nimte.ac.cn (C.Y); linzhengde@nimte.ac.cn (C.-T.L)

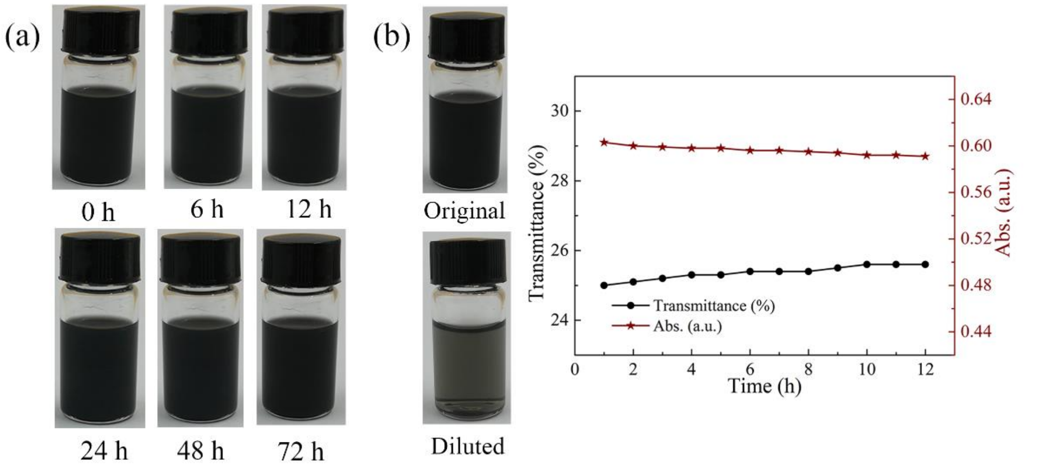

Figure 1. (a) The storage stability test of CNTs/AgNWs dispersion in ethanol at room temperature. (b) The transmittance and absorbance of the dispersion as a function of standing time.

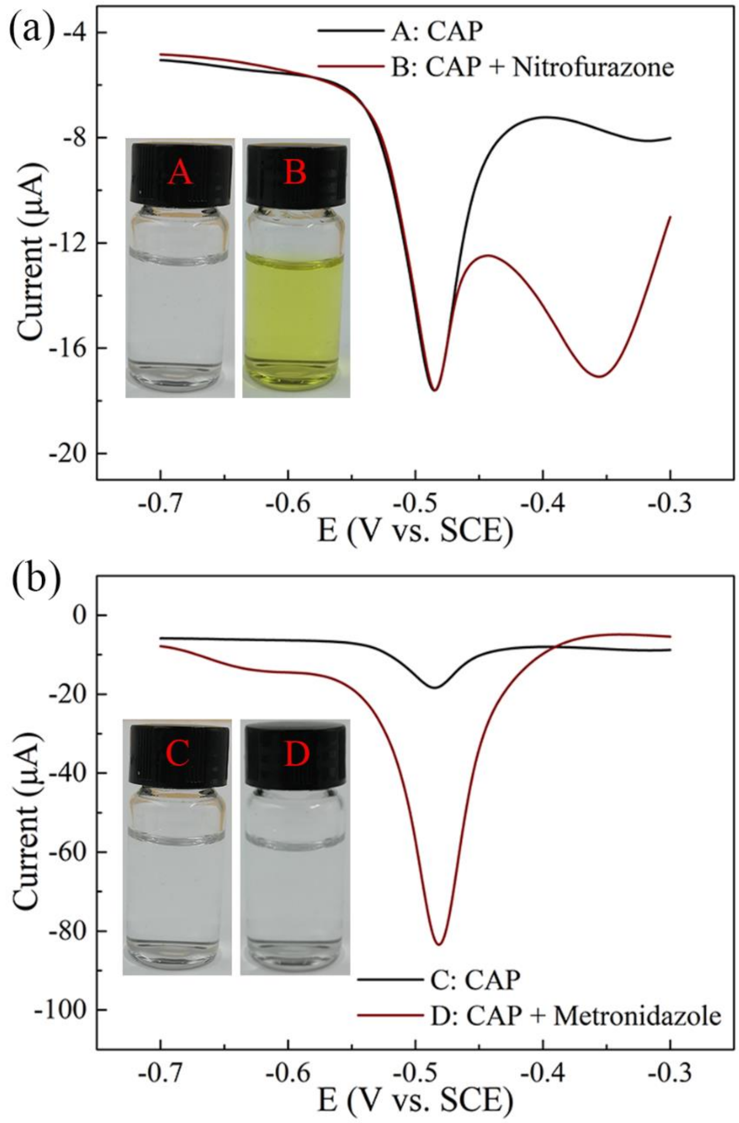

Figure 2. Interference study of CAP detection in the presence of Nitrofurazone (**a**), and Metronidazole (**b**).

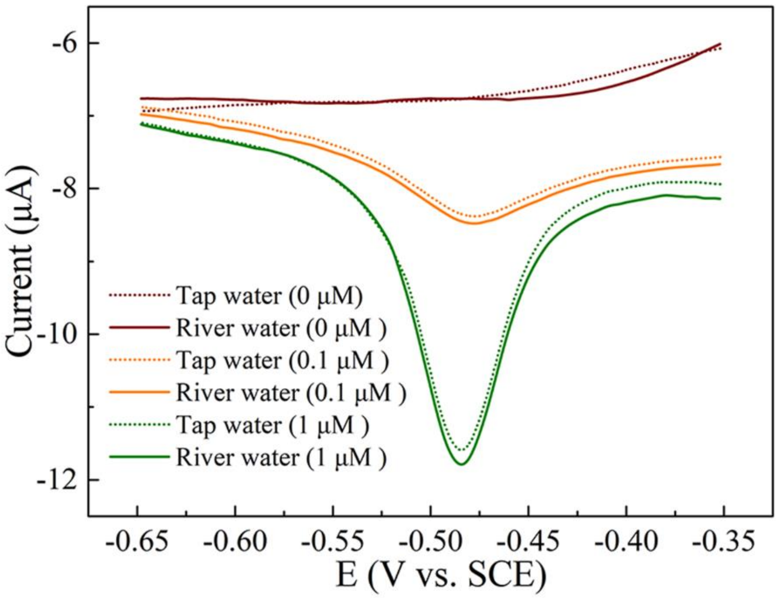

Figure 3. DPV curves of CNTs/AgNWs electrodes for real samples analysis.
